# Mapping the evidence for the prevention and treatment of eating disorders in young people

**DOI:** 10.1186/2050-2974-2-5

**Published:** 2014-02-03

**Authors:** Alan P Bailey, Alexandra G Parker, Lauren A Colautti, Laura M Hart, Ping Liu, Sarah E Hetrick

**Affiliations:** 1 Orygen Youth Health Research Centre, Centre for Youth Mental Health, The University of Melbourne, Locked Bag 10, Parkville, Melbourne, Victoria 3052, Australia; 2 Centre of Excellence in Youth Mental Health, headspace National Youth Mental Health Foundation Ltd, Level 2, South Tower, 485 La Trobe Street, Melbourne, Victoria 3000, Australia; 3 Melbourne School of Population Health, The University of Melbourne, Level 3, 207 Bouverie Street, Melbourne, Victoria 3010, Australia

**Keywords:** Eating disorders, Treatment, Prevention, Evidence mapping, Adolescent, Young adult

## Abstract

**Abstract:**

Eating disorders often develop during adolescence and young adulthood, and are associated with significant psychological and physical burden. Identifying evidence-based interventions is critical and there is need to take stock of the extant literature, to inform clinical practice regarding well-researched interventions and to direct future research agendas by identifying gaps in the evidence base.

**Aim:**

To investigate and quantify the nature and distribution of existing high-quality research on the prevention and treatment of eating disorders in young people using evidence mapping methodology.

**Method:**

A systematic search for prevention and treatment intervention studies in adolescents and young adults (12–25 years) was conducted using EMBASE, PSYCINFO and MEDLINE. Studies were screened and mapped according to disorder, intervention modality, stage of eating disorder and study design. Included studies were restricted to controlled trials and systematic reviews published since 1980.

**Results:**

The eating disorders evidence map included 197 trials and 22 systematic reviews. Prevention research was dominated by trials of psychoeducation (PE). Bulimia nervosa (BN) received the most attention in the treatment literature, with cognitive behavioural therapy (CBT) and antidepressants the most common interventions. For anorexia nervosa (AN), family based therapy (FBT) was the most studied. Lacking were trials exploring treatments for binge eating disorder (BED) and eating disorder not otherwise specified (EDNOS). Relapse prevention strategies were notably absent across the eating disorders.

**Conclusions:**

Despite substantial literature devoted to the prevention and treatment of eating disorders in young people, the evidence base is not well established and significant gaps remain. For those identified as being at-risk, there is need for prevention research exploring strategies other than passive PE. Treatment interventions targeting BED and EDNOS are required, as are systematic reviews synthesising BN treatment trials (e.g., CBT, antidepressants). FBTs for AN require investigation against other validated psychological interventions, and the development of relapse prevention strategies is urgently required. By systematically identifying existing interventions for young people with eating disorders and exposing gaps in the current literature, the evidence map can inform researchers, funding bodies and policy makers as to the opportunities for future research.

## Introduction

Adolescence and young adulthood is recognized as a period of heightened risk for the development of eating disorders. International epidemiological studies estimate 75% of anorexia nervosa (AN) and bulimia nervosa (BN) cases and 50% of binge eating disorder (BED) and eating disorder not otherwise specified (EDNOS) cases onset before the age of 22 [1,2]. Eating disorders are recognised as a significant public health issue with Australian data indicating that they represent the second leading cause of disability due to mental disorder in females aged 10–24 years [3]. The psychological, social and physical ramifications of eating disorders are severe [4,5]. A recent meta-analysis showed mortality rates to be twice as high in those with BN and EDNOS and close to six times higher in people with AN when compared to expected population mortality rates [6]. Suicide contributes significantly to these high mortality rates, with rates of suicide elevated among those with eating disorders [7-9]. Comorbidity with depression, anxiety and substance use disorders is common [10-13], adding significantly to the burden. Additionally, the estimated financial cost associated with disability-adjusted life years attributable to eating disorders is higher than that of depression and anxiety combined [14].

Given the severity and burden associated with eating disorders, and the particular vulnerability across the adolescent and young adult period, there is pressing need to both develop, and encourage the uptake of, evidence-based prevention and intervention strategies with this population. Despite the need, and consistent with mental health research generally [15], evidence-based interventions are far from universally delivered in the clinical management of eating disorders [16-19]. For example, cognitive behavioural therapy (CBT) is recognised as an empirically supported intervention and the ‘treatment of choice’ for BN [20-22], yet surveys of clinicians indicate a majority do not use CBT as their primary psychological treatment choice [23,24]. This highlights a significant evidence-practice gap.

A commonly cited barrier to incorporating evidence into daily clinical practice is the time and resources required to navigate the large volume of extant research literature (e.g., [25]). This points to the need for translational tools that make these vast bodies of literature accessible, digestible and usable. One such approach to knowledge translation and exchange is evidence mapping; an established methodology for collating and summarising the literature in a manner that enables the breadth of the research activity in a particular field to be explored (e.g., [26-28]). Evidence maps are based on an explicit research question relating to the field of enquiry, which drives the search for, and collection of, appropriate studies utilising explicit, systematic and reproducible methods [29-32]. The key difference from a systematic review is that the purpose of evidence mapping is to provide a broad overview of existing research, with the view to identifying evidence, and therefore does not include an in-depth quality appraisal and synthesis of the findings [30]. The end-user may be: 1) Researchers or research funding bodies who can utilise the gaps in the evidence highlighted in the evidence map to drive their research agenda; 2) Policy makers who can use the evidence map to inform policy decisions; and 3) Clinicians who can quickly and easily access information about interventions.

This paper presents the results of an evidence map we conducted on eating disorders in adolescents and young adults. The extent, range and nature of high-quality clinical research interventions for eating disorders in this population is summarised, gaps in the evidence base are identified and opportunities for future research are discussed. This process of taking stock of the evidence is an essential first step in collating the breadth of research activity in an area before further exploring the effectiveness of interventions.

## Methods and materials

The eating disorder evidence map was produced as part of a larger evidence-mapping project undertaken by the Centre of Excellence (CoE) in Youth Mental Health (part of headspace; the Australian National Youth Mental Health Foundation). A detailed description of the methodology for evidence mapping has been published elsewhere [31]; methods specific to the eating disorder map are provided below.

### Creating a map based on broad clinical questions relating to the field of enquiry

After consultation with expert youth mental health researchers and clinicians working within the eating disorders field at Orygen Youth Health Research Centre and headspace, the questions and scope of the mapping project were defined. This process revealed two critical questions:

i. What evidence exists regarding interventions for eating disorders in the youth population?

ii. What areas are, and are not, well researched?

#### Defining key variables, specifying characteristics to be mapped and developing inclusion and exclusion criteria

Based on these explicit questions, the characteristics of studies to be included in the map were defined, which included specifying the population, stage of eating disorder, measured outcomes and study design.

#### Population

Included trials were required to have participants with a mean age between 12 and 25 years as their sample, or where the author specified an adolescent and/or young adult population. Studies with both adult and adolescent participants were included if the mean age of any intervention group was 25 years or under. Studies that recruited participants on the basis of physiological or medical conditions (e.g., changes in eating behaviours induced by brain tumour) were excluded.

#### Stage of eating disorder and outcomes

Prevention, treatment intervention and relapse prevention studies were all included. Prevention trials were categorised as universal or at-risk prevention. Universal interventions were those delivered to a designated population regardless of their risk (e.g., all school students); whereas at-risk prevention interventions were those delivered to either members of a population with an established risk factor for the development of an eating disorder (e.g., athletes) or individuals with signs or symptoms of a disorder such that participants were recruited on the basis of elevated risk factors or sub-threshold presentations of an eating disorder [33].

Studies including those with an established eating disorder diagnosis, classified by the diagnostic and statistical manual of mental disorders (DSM) [34] or the international classification of diseases (ICD) [35], were included where the treatment intervention was "anything delivered for the purpose of relieving symptomatology or improving functioning of the target disorder" [31]. These intervention studies were classified as ‘established eating disorder’.

Relapse prevention studies were included if the authors specified the intervention was designed to prevent relapse, or to maintain improvements in individuals with eating disorders who had previously responded to treatment.

Finally, included trials had to measure a specific eating disorder outcome, for example body mass index (BMI), eating disorder symptom measure, binge/purge frequency.

#### Study design

Our map utilised evidence from randomised controlled trials (RCT), controlled clinical trials (CCT), systematic reviews and meta-analyses, as these are considered the most robust study designs for examining the effectiveness of interventions [36]. Definitions of review types are not consistent and many different terms are used, at times interchangeably [29], therefore we included those reviews providing both a systematic search strategy and the relevant data-bases searched. Additionally reviews had to meet the criteria outlined above. Studies published in English from 1980 to December 2012 were included.

#### Searching the literature

Search strategies appropriate for each of the MEDLINE, PSYCINFO and EMBASE databases were devised using terms such as "eating disorder", "controlled trial" and "review". Additional terms identified by experts were also included.

Searches targeted the pre-defined inclusion criteria by combining terms describing: type of eating disorder, study methodology and stage of eating disorder (the full search strategy is available upon request to the corresponding author). Search strategies were revised after a random sample of 100 citations was examined and after cross-checking that the search strategy retrieved 20 articles known to meet the inclusion criteria.

#### Screening and positioning the relevant evidence within the map (charting)

Two authors independently screened 100 references randomly selected from the search results to determine the consistency of applying the inclusion/exclusion criteria (inter-rater reliability > 0.90 was achieved). Two authors then screened all retrieved references and where a title or abstract reported a trial that appeared to be eligible for inclusion, the full article was obtained and assessed against the inclusion and exclusion criteria.

Studies meeting the inclusion criteria were coded by one author and double coded independently by a second author. Discrepancies were discussed and consensus reached. Studies were coded according to: 1. Type of eating disorder; 2. Type of intervention; 3. Stage of eating disorder; and 4. Study design. Type of eating disorder was coded as AN, BN, EDNOS or BED, according to author report of participant classification. Type of intervention was classified as psychological, biological and service/delivery improvement. Stage of eating disorder was coded as universal prevention, at-risk, established eating disorder and relapse prevention. Study designs were coded as RCT/CCT (hereafter referred to as ‘trials’) and systematic review/meta analysis (hereafter referred to as ‘reviews’).

Studies that evaluated the effectiveness of more than one type of intervention or eating disorder classification were double coded for each intervention and classification, thus the sum of trials from each coded section is greater than the total number of included studies. The primary reference for each study was established with secondary publications indicated as such. This process prevented counting one trial multiple times and misrepresenting the number of studies in a particular area. In addition to these codes, a qualitative description of the interventions used in each trial was recorded. For the purposes of this paper, where a review was available and included in the map, a short synthesis of the main findings has been provided.

## Review

### Included trials

**Figure 1:**
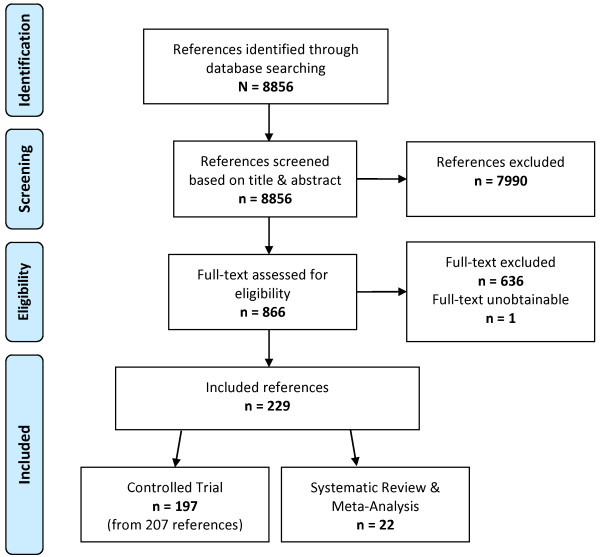
Flow chart of included studies.

Database searches were conducted in February of 2013 and our strategies identified 8,856 references, of which 229 met the predefined inclusion criteria based on title, abstract and full text screening (see Figure 1). The 229 publications included in the final map consisted of 22 reviews, 197 trials and 10 follow-up studies. A list of citations for all included studies in the map is available on request or from our online searchable database (see [37]).

### Interventions for prevention

A total of 52 universal prevention studies were identified (46 trials; 6 reviews). Of the 46 trials, psychoeducation based programs were the most common (n = 26), followed by cognitive dissonance (n = 6) and cognitive behavioural based (n = 5) programs. The remaining trials investigated non-specific psychological strategies such as media literacy training, writing tasks and school-based prevention programs.

A total of 46 prevention studies for at-risk populations were identified (40 trials; 6 reviews). Of the trials, psychoeducation based (n = 12) and cognitive dissonance based (n = 12) programs were most common, followed by cognitive-behavioural based programs (n = 7). Other non-specific psychological intervention trials included multicomponent school based programs, media literacy training, writing tasks, relaxation and yoga. Table 1 displays the complete distribution of identified studies for prevention in both universal and at-risk populations.

**Table 1:** Distribution of included prevention studies

	**Intervention**	**Trial**	**AN**	**BN**	**BED**	**EDNOS**	**ED**	**Review**
**Universal prevention**								
Psychological	Cognitive dissonance	6					6	
	Psychoeducation based	26					26	
	Cognitive/Behavioural based	5					5	1
	Psychological - other	14	1				13	1
	Psychological - general							4
**At-risk prevention**								
Psychological	Cognitive dissonance	12					12	
	Psychoeducation based	12		2			10	
	Cognitive/Behavioural based	7		1			6	1
	Motivational interviewing	1		1	1	1		
	Psychological - other	18		3	2		13	1
	Psychological - general							4

**Legend:** AN=anorexia nervosa, BN=bulimia nervosa, BED=binge eating disorder, EDNOS=eating disorder not otherwise specified, ED=eating disorder general.

Reviews summarising prevention programs for both universal and at-risk populations indicate the evidence for their effectiveness is modest and not without significant limitations [38-43]. Meta-analyses of controlled trials indicate prevention programs generally produce large effects on outcomes related to eating disorder knowledge, and only small net effects for other important prevention targets such as reducing exhibited risk factors, changing attitudes and reducing eating pathology. This has prompted the search for and identification of moderating factors that may lead to larger prevention effects, for example targeting high-risk populations versus universal, utilising active programs delivered in interactive formats versus passive didactic programs (e.g., psychoeducation) and multisession programs versus single session (see [39,42]). Promising preventative effects are generally exhibited directly following program delivery and over the immediate short-term, however little work has been done investigating whether effects are sustained long-term. This lack of follow-up testing combined with the limited use of standardised eating pathology measures and diagnostic criteria makes it difficult to accurately conclude on the preventative effects of most evaluated programs.

### Interventions for an established eating disorder

A total of 129 studies investigating interventions for established eating disorders were identified (see Table 2), of which 77 involved psychological interventions (67 trials; 10 reviews), 54 involved biological interventions (47 trials; 7 reviews).

**Table 2:** Distribution of included disorder established treatment studies

	**Intervention**	**Trial**	**AN**	**BN**	**BED**	**EDNOS**	**Review**
**Psychological**	Cognitive & behavioural therapies	27	3	22	2	5	
	Psychoeducation	10	6	6			
	Family based therapy (FBT)	15	12	5		2	5
	Supportive therapy	3	1	3			
	Psychodynamic/Psychoanalysis	5	1	3			1
	Motivational interview/enhancement	4	2	3	1	3	
	Exposure therapy	2		2			
	Interpersonal therapy	2		2			
	Dialectical behavioural therapy	1		1			
	Eye movement desens. & reprocessing	1	1	1		1	
	Psychological - other	27	14	18	2	6	
	Psychological - general						4
							
**Biological**	SSRI	15	4	9	1	1	
	TCA	10	3	8			
	SNRI	1				1	
	MAOI	1		1			
	Appetite moderator	6	1	5	1		
	Anticonvulsant	1		1			
	Typical antipsychotic	1	1				2
	Atypical antipsychotic	6	6			1	3
	Lithium	2	1	1			
	Antidepressant - other (trazodone)	1		1			
	Opioid antagonist (naltrexone)	1		1			
	Neutraceutical (inositol)	1		1	1		
	Transcranial magnetic stimulation	1		1			
	Re-feeding	2	2				
	Biological - other	4	3	1			
	Biological - general						4
							
**Service**	Service/Delivery improvement	6	3	4		1	

**Legend:** AN=anorexia nervosa, BN=bulimia nervosa, BED=binge eating disorder, EDNOS=eating disorder not otherwise specified, SSRI=selective serotonin reuptake inhibitor, TCA=tricyclic antidepressant, SNRI=serotonin norepinephrine reuptake inhibitor, MAOI=monoamine oxidase inhibitor.

### Psychological interventions for established eating disorders

Of the identified studies investigating psychological interventions, the most common strategy for diagnosed AN was family based therapy (FBT, 12 trials; 5 reviews). For diagnosed BN, trials were dominated by cognitive behavioural therapy (CBT, n = 22) with a smaller number investigating FBT (n = 5). Psychological intervention trials for EDNOS and BED were scarce in comparison. Table 2 displays the complete distribution of identified studies by type of psychological intervention and type of disorder.

Included reviews of psychological interventions for diagnosed eating disorders were restricted to syntheses of family based therapies (FBT) for AN, with one review also including BN [44-50]. FBT as defined in this paper and summarised in included reviews refers to any intervention where the family constitutes a core intervention component. The most commonly evaluated models include the Maudsley Model [51] and its variants and a highly similar modality called behavioural family systems therapy [52]. Reviews highlight the growing evidence base for FBT in AN, providing summaries of RCTs which support its position in clinical guideline recommendations as a first line treatment. Evidence indicates that FBTs are generally effective with reviews indicating up to half of adolescents receiving the intervention achieving remission. A smaller subset of studies has noted higher rates of remission at follow-up for FBT compared to individual level interventions. FBT also appears to be more effective for younger adolescents and those with a shorter duration of illness, however the majority of trials only report mean participant ages in the 12–18 range. It should be noted that these findings come only from a small number of trials with small sample sizes, where risk of bias is notable. Nonetheless, syntheses from the included reviews provide mostly supportive conclusions for FBT as an effective treatment for AN in young people.

A single recently published review of FBTs investigated this modality in BN [47], summarising two extant RCTs. The first trial found higher remission rates for FBT versus individual supportive therapy at post-treatment and 6 months [53]; the second trial found higher binge abstinence rates for CBT guided self-care versus FBT at post-treatment but no differences at 6 months [54]. FBT is promising but requires further verification in BN before stronger recommendations about its effectiveness can be made. No reviews of other psychological interventions for BN were identified. Similarly reviews for BED and EDNOS were absent.

### Biological interventions for established eating disorders

Of the identified studies investigating biological interventions, medications were the most common. For diagnosed BN, selective serotonin reuptake inhibitors (SSRI, n = 9) and tricyclic antidepressants (TCA, n = 8) were the most investigated. For diagnosed AN, antipsychotics (7 trials; 3 reviews) were the most common, followed by SSRIs (n = 4). Biological interventions for EDNOS and BED were almost non-existent. Table 2 displays the complete distribution of identified studies by type of biological intervention and type of disorder.

Included reviews synthesised findings from controlled medication trials and were restricted to investigations of antipsychotics in AN [55-57] and general medication reviews including analyses of antidepressants for AN and BN [58,59]. Reviews of controlled medication trials for EDNOS and BED in our population were absent.

Antipsychotics for AN were synthesized by meta analysis (mean age 22 years across 8 RCTs [56]), with findings indicating no advantage over placebo or treatment as usual in terms of weight gain or symptom improvement.

General medication reviews did not identify any trials investigating antidepressants in adolescents (12–18 years) [58,59]. While trials exist in the younger adult population (18–25 years), these were lost within broad adult level syntheses. For adult AN, the use of both TCAs and SSRIs has not produced encouraging results [58], with safety issues and lack of effectiveness data making translation to younger populations problematic. For adult BN, SSRIs have produced positive findings with moderate effects on binge/purge frequency and reviews indicate their potential utility with young people [58,59]. However this needs to be balanced with the lack of current efficacy and safety data in this younger population, particularly given the controversy surrounding the use of SSRIs with adolescents and young adults in the depression field (see [60]). The lack of trials in adolescents and the urgent need to systematically synthesise existing young adult trials means medication management recommendations are currently restricted to broad adult level evidence.

### Interventions for relapse prevention

Research into relapse prevention among young people with eating disorders is limited. Only six trials were identified; five trials in AN (n = 3 CBT; n = 1 psychoeducation; n = 2 SSRI) investigating relapse prevention strategies following successful inpatient weight restoration, and one trial in BN investigating SSRIs delivered after successful psychotherapy (see Table 3). No systematic reviews were identified.

**Table 3:** Distribution of included relapse prevention studies

	**Intervention**	**Trial**	**AN**	**BN**	**BED**	**EDNOS**	**Review**
**Psychological**	Cognitive & behavioural therapies	3	3				
	Psychoeducation	1	1				
							
**Biological**	SSRI	3	2	1			

**Legend:** AN=anorexia nervosa, BN=bulimia nervosa, BED=binge eating disorder, EDNOS=eating disorder not otherwise specified, SSRI=selective serotonin reuptake inhibitor.

## Discussion

The results of the evidence map highlight areas that have received research attention while simultaneously exposing numerous opportunities for research in the area of eating disorder intervention. Gaps in the current evidence base are discussed with a focus on specific research opportunities.

### Research opportunities for prevention

Prevention literature is heavily dominated by education based programs and while providing information on etiology and risk does not produce iatrogenic effects [41], these programs are minimally effective in producing behaviour change, outside of increasing knowledge [40]. There are suggestions that programs utilising active prevention components may produce larger effects and many opportunities exist [39,42].

Cognitive dissonance or dissonance induction is one such active prevention approach which involves engaging in counter-attitudinal exercises that target potential risk factors for eating pathology (e.g., body dissatisfaction and internalisation of the ‘thin-ideal’) [61]. Research to date indicates these interventions show promise in changing attitudes associated with eating pathology over the short term [40-42], however further trials involving long term follow-up are need to determine if lasting effects on standardised eating pathology outcomes are possible. Media literacy programs have also shown promise and incorporating active dissonance exercises and simple CBT techniques has the potential to boost preventative effects.

Interestingly, our map failed to identify any controlled trials investigating prevention strategies involving families. This is curious given eating disorders often develop in adolescence; a time for many when the family structure is critical to functioning. Potential prevention components may involve education and skills-training for parents around talking about and identifying risk factors. Such components may assist in creating awareness, identifying the early stages of disordered eating or other symptomatology, ensuring parents have some control over their child’s eating (particularly in those identified as at-risk) and importantly encouraging early help-seeking.

### Research opportunities for those with established eating disorders

#### Systematic reviews

The evidence map revealed a paucity of systematic reviews summarising a range of treatment options for young people across the eating disorders. For example, many trials have now been conducted investigated interventions for BN such as CBT (22 trials) and SSRIs (9 trials) yet systematic reviews of these intervention modalities are absent. Results from individual trials are mixed regarding the effectiveness of either intervention, suggesting that submitting the trials to systematic review and meta-analysis may provide a summary answer to the effectiveness question. While both CBT and SSRIs have received attention in the adult literature, with systematic reviews demonstrating the clinical effectiveness of these approaches [21,62], the comprehensive synthesis of adolescent and young adult trial data would provide important clinical guidance about the appropriateness and effectiveness of these interventions in this younger population.

#### Stepped-care models of intervention

Stepped care for the management of mental disorders is not a new concept (e.g., [63-65]) yet these intervention models have received limited empirical attention in the field of eating disorders. Our map identified two trials investigating stepped-care models for the treatment of BN in young people [66,67]. Both trials compared a self-help manual with standard CBT in the initial phase, with those failing to respond being offered modified CBT in one trial [67] and fluoxetine plus CBT (if still unresponsive) in the second trial [66]. Both trials indicated that a stepped-care model was as effective as the main CBT intervention delivered, indicating a potentially lower cost/less resource intensive intervention model can produce similar effects.

Stepped care approaches typically commence with low-intensity treatment components and step up, depending on response and stage of illness, to more intensive, individualised, clinician delivered strategies. These are considered advantageous as they can be cost effective in a resource limited field, reduce delay-to-treat time by offering low-intensity components such as self-help as an initial step, and can be tailored to individual treatment plans according to illness severity and treatment response [65,68,69].

While these models do show promise, little can be definitively concluded about the effectiveness and appropriateness of stepped care interventions for young people with eating disorders. Significant questions remain regarding how decisions are made about progressing through intervention ‘steps’ (e.g., using response and/or stage of illness); appropriateness in younger age groups; how those who do not respond should be managed; and whether these models are appropriate for high severity disorders like AN which typically require intensive, sustained intervention [69]. Given these remaining questions and the potential benefits associated with these models, further research attention is warranted.

#### Binge eating disorder

Intervention research for BED among the adolescent and young adult population is lacking. Reviews of the adult literature point to CBT as a promising intervention in reducing binge frequency and other associated body image concerns, however changes in BMI are yet to be noted [70,71]. The map identified only two small trials of CBT in the adolescent and young adult population and while some positive effects on bingeing behaviour were noted [72,73], it remains difficult to draw conclusions about its appropriateness and effectiveness.

The promising nature of CBT warrants further investigation in the context of a much greater research effort directed towards BED given it is a stable and chronic syndrome, with serious physical and psychological ramifications including a heightened risk for obesity [74-76]. Despite a marginally later onset than other eating disorders, BED does occur in the younger population [1,2] highlighting the importance of developing effective interventions, particularly as adolescence represents a critical period for the development and maintenance of positive health behaviours [77].

While we acknowledge that the lack of intervention studies likely reflects BED’s previous status as a research category according to DSM-IV-TR [78], it is anticipated that its inclusion as a diagnostic category in DSM-5 [79] will provide impetus for further intervention specific research in this area.

#### Family based therapy

Intervention research is dominated by trials of family based therapy, particularly for AN in the adolescent bracket. Even with the widespread implementation of FBTs due to clinical practice guideline recommendations (e.g., [20,80]), further well-conducted trials are still required, not only to provide a stronger evidence base for clinical recommendations, but to address significant clinical questions that remain. For example, is FBT the most effective psychological intervention for AN? Head to head trials with other validated psychological interventions adapted for the eating disorders are required. CBT is a potential candidate given its success in BN and the evidence base generated across other adolescent/young adult onset disorders. Interpersonal therapy also presents an interesting comparison as its relationship framework may have overlapping features with FBT. Important work is also needed to determine the effectiveness of FBT in populations other than adolescents. A recent review described developments of an adapted version of FBT for young adults (18+) [47]. The main adaptation being the definition of family where a pragmatic approach is used to define a ‘family of choice’ which may include partners, close friends, room mates etc. This approach has the potential to make an effective intervention available to a wider population.

#### Eating disorder not otherwise specified

The evidence map highlights the absence of intervention studies investigating EDNOS treatments in adolescents and young adults. This is concerning given that data suggest more than 50% of those diagnosed with an eating disorder have EDNOS [81,82] and it being the most common diagnosis reported in clinical and community populations with eating disorders [83,84]. Additionally, those with EDNOS follow a similar course to those diagnosed with AN and BN in terms of the nature, symptom severity, eating pathology and outcome of disorder [85-87].

Due to the similarities EDNOS shares with AN and BN, and problems recruiting sufficient sample sizes in eating disorder research [88], it can be common for researchers to include participants with EDNOS within trials investigating interventions for AN or BN, either by including EDNOS diagnosis or by using relaxed AN/BN inclusion criteria which facilitates entry of EDNOS participants. This may explain the lack of trials specifically targeting those with EDNOS, potentially masking the evidence base for effective EDNOS intervention. However, only a small number of trials explicitly reported doing so and those that did failed to provide the adequate sub-group analysis required to determine intervention effects according to eating disorder diagnosis.

Given the similarities between EDNOS and AN/BN categories and in line with guideline recommendations indicating treatment of EDNOS follow the disorder-symptom profile most closely resembled (either AN or BN [20]), those treatments which have previously shown promise in the AN and BN area (e.g., FBT, CBT) should be prioritised in future EDNOS intervention research.

### Research opportunities for relapse prevention

While rates of relapse have yet to be systematically investigated, longitudinal cohort and treatment follow-up studies estimate 20% to 50% of those with eating disorders will relapse [89-94]. Given these high relapse rates and the burden associated with a relapsing and chronic long-term condition, as is common among those with eating disorders, effective relapse prevention strategies are vital. The lack of trials investigating relapse prevention in adolescents and young adults with eating disorders is one of the most salient findings of this evidence map.

Three small trials were identified utilising CBT as a relapse prevention strategy with post-hospitalised, weight restored AN participants, showing positive preliminary results [95-97]. SSRIs were also investigated in another two trials using similar weight restored samples, however no effects above placebo were noted [98,99]. Of concern is that large proportions of intervention arm participants across these trials still experienced relapse.

CBT-based relapse prevention strategies appear promising, as noted in the above trials and highlighted in the adult AN literature [100], but require further investigation. Newer ‘third wave’ strategies like mindfulness, which has shown some degree of success in adults with depression [101,102], are now beginning to receive attention in the eating disorders literature (e.g., [103-105]) and similarly require investigation in younger populations. Given the age of onset of most eating disorders [1,2], it is interesting that family-based relapse prevention strategies have not yet been developed. Such strategies are noted in other high severity disorders affecting young people, for example first episode psychosis [106] and bipolar disorder [107]. Once a young person leaves primary treatment, they return to an environment that is potentially unchanged, where significant risk factors remain [108]. The family, and thus family-based interventions, may be well positioned to address factors associated with relapse and assert an important influence in maintaining behaviour change and preventing or minimising relapse.

### Limitations

Syntheses of intervention effects were not undertaken, restricting us from drawing conclusions on intervention efficacy. Additionally, study quality appraisal was not conducted; therefore studies with methodological flaws may still be included. Furthermore, included studies were restricted to ‘gold standard’ research, which neglects important information available from studies utilising designs other than controlled trials and systematic reviews. For example, research areas where it may be unethical to randomise patients to particular conditions (e.g., those with chronic and severe disorders) would be absent from the evidence map. Nonetheless, this evidence map has systematically demonstrated what prevention and treatment interventions exist and has exposed important gaps in the current literature.

## Conclusions

The onset of eating disorders frequently occurs in adolescence and young adulthood and is associated with significant psychological and physical burden. While promising interventions for this population have been identified, gaps exist and the body of evidence has not firmly established which interventions are most effective. Systematic reviews are required in some areas where the evidence base has yet to be summarised in a meaningful way (e.g., the use of CBT and antidepressants for diagnosed BN). Interventions which require further investigation in well conducted controlled trials include prevention programs for those at risk of developing eating disorders, focusing on intervention components other than passive psychoeducation; head-to-head trials of FBTs and other validated psychotherapies for AN; investigation of stepped-care intervention models; psychological interventions for BED and EDNOS; and interventions designed to prevent relapse or maintain treatment response. By systematically identifying existing interventions for young people with eating disorders and exposing gaps in the current evidence base, this evidence map can inform researchers, funding bodies and policy makers as to the opportunities for future research.

## Abbreviations

AN: Anorexia nervosa; BN: Bulimia nervosa; EDNOS: Eating disorder not otherwise specified; BED: Binge eating disorder; FBT: Family based therapy; CBT: Cognitive behavioural therapy; PE: Psychoeducation; SSRI: Selective serotonin reuptake inhibitor.

## Competing interests

The authors declare that they have no competing interests.

## Authors’ contributions

APB contributed to screening and coding of included articles and drafted the manuscript, AGP participated in the design of the study and helped draft the manuscript, LAC participated in screening and coding of included studies and helped draft the manuscript, LMH provided advice and helped draft the manuscript, PL participated in screening and coding of included studies and helped draft the manuscript, SEH conceived the study, participated in its design and helped draft the manuscript. All authors read and approved the final manuscript.
